# Osteosynthesis-screw augmentation by ultrasound-activated biopolymer - an ovine *in vivo* study assessing biocompatibility and bone-to-implant contact

**DOI:** 10.1186/s13018-015-0156-1

**Published:** 2015-01-28

**Authors:** Hanjo Neumann, Stefan Breer, Nils Reimers, Richard Kasch, Arndt-Peter Schulz, Benjamin Kienast

**Affiliations:** BG Trauma Hospital, Hamburg, Germany; Medical Faculty, University of Lübeck, Lübeck, Germany; Department of Orthopaedics, University Greifswald, Greifswald, Germany

**Keywords:** Biocompatibility, Ultrasound-activated polymer, Augmented screw, Ovine *in vivo* study

## Abstract

**Objectives:**

Screw fixation and fragment anchoring in osteoporotic bones is often difficult. Problems like the cut out phenomenon and implant migration in osteoporotic bones have been reported. One possibility of improving the anchoring force of screws is augmentation of the screw. Cement-augmented screws in spinal surgery could exhibit a better anchoring in osteoporotic bones.

**Methods:**

The purpose of this study was to examine the effect of screw augmentation using a resorbable polymer. Ultrasound-activated biodegradable pins were used for the purpose of a resorbable augmentation technique. Cannulated screws were inserted into the femur of 12 sheep and augmented by an ultrasound-activated polylactic acid (PLDLA) pin. In a paired approach, four screws were implanted in each animal: 2× a 10-mm thread and 2× a 20-mm thread, both of which were augmented with polymer. Both screws, named A and B, were also applied without augmentation (control group) and implanted into the contralateral hind limb. After 4, 8, and 12 weeks, the sheep were euthanized and a macroscopical and histological examination followed.

**Results:**

The polymer spread well out of the screws into the cancellous lacunae. Around the polymer, the peripheral bone showed signs of healthy and active bone tissue. No evidence of inflammation or infection was observed. The boneto-implant contact was significantly higher in the augmented screws. Biocompatibility was proven in histopathological examination. After 12 weeks, no pathological changes were found.

**Conclusion:**

Ultrasound-activated polymer augmentation of cannulated screws may improve the anchoring in osteoporotic bone.

**Article focus:**

- Can screw augmentation using a resorbable polymer improve the bone-to-implant contact in case of screw osteosynthesis?
- Is there any effect on the surrounding tissue by the induced temperature and liquefied polymer?
- Can biocompatibility be proven by this new osteosynthesis?

**Key messages:**

- Screw augmentation by ultrasound-activated biopolymer leads to a significant higher bone-to-implant contact than pure screw osteosynthesis.
- No tissue damage could be observed by the application of the SonicFusion™.

**Strength and limitations of this study:**

- The ovine *in vivo* study concept can simulate physiological conditions.
- First examination of screw augmentation by ultrasound-activated biopolymer.
- No biomechanical testing of the higher bone-to-implant contact by now.

## Introduction

The number of osteoporotic fractures has risen dramatically in the last two decades and demographic data shows a further increase of these injuries [1,2]. Screw fixation and fragment anchoring in osteoporotic bones can be extremely difficult [3-5]. Problems like the cut out phenomenon and implant migration in osteoporotic bones with the need of revision operations have been reported [3,6].

Therefore, many different solutions are currently under investigation. The design of the osteosynthesis screw has changed from a round profile to a blade design to prevent a migration of the implant in the osteoporotic bone [7]. Another approach to a stronger fixation of screws in osteoporotic bones is the preparation of the implant surface with bioactive substances. In a study with rats, Zoledronate-coated screws exhibit a higher bone density around the implant [8]. Calcium pyrophosphate-coated titanium screws exhibited a significantly higher toque when removing the screw after healing compared to uncoated screws [9].

Another idea for improving the anchoring force of screws is the augmentation of the screw itself [10,11]. Over the past years, augmentation of screws with bone cement (poly(methylmethacrylate) (PMMA)) was intensively investigated in the area of spinal surgery. The pullout force of screws in the pedicle proved to be higher with PMMA cement augmentation [12,13]. Newer studies showed similar pullout forces using calcium phosphate cement [14]. In comparison to PMMA cement, the solidification of calcium phosphate cement is non-exothermic with a lower potential for thermal necrosis of the adjacent tissue, and most importantly, it is bioresorbable [15]. *In vivo* studies and biomechanical studies report a leakage rate of 30%–50% [16,17]. A typical problem in the osteoporotic bone of the typical elderly patient is the cut out phenomenon, *when intramedullary osteosynthesis devices can perforate the cortex of the bone and lead to complications* [18]. Cement augmentation of an intramedullary osteosynthesis in laboratory analysis was able to prove an enhanced pullout resistance and a stronger rotational stability [10,19,20]. Clinical experience in cement-augmented osteosynthesis for femoral neck fractures showed promising results [21,22].

The aim of this study was to examine screw augmentation with an absorbable polymer. In recent times, ultrasound-activated biodegradable pins have been tested for the purpose of being used for resorbable osteosynthesis [23-27]. The biocompatibility and safety of these (non-activated) co-polymers has been demonstrated in several studies [28,29]. The examination of ultrasound-activated pins showed no specific tissue damage caused by the induced heat of the melting process. There was rather evidence of an enhanced bone/implant contact [23].

During the study, the *in vivo* biocompatibility and performance of the so-called “SonicFusion™” inside-out technology was tested using a sheep model. Therefore, cannulated screws were augmented by an inserted, ultrasound-activated polylactic acid (PLDLA) pin, which can melt through perforations in the screw into the surrounding tissue. This study shall prove a higher bone-to-implant contact (BIC) of a screw osteosynthesis by augmentation of the screw with an ultrasound-activated PLDLA pin and shall show that the melting process causes no further damage to the surrounding tissue.

## Material and methods

### Animals

Twelve female sheep (*Ovis aries*), at least 2 years of age (adult animals), were used for this examination. At time of surgery, their mean weight was 59.3 kg. The acclimation period was 5 days; 24 h before surgery, the animals started fasting.

This study was conducted in accordance with the requirements of the FDA Good Laboratory Practice (GLP) regulations, 21 CFR 58 (revision of 1 April 2007) and the OECD Good Laboratory Practice, reference ENV/CM/CHEM (98)11 adopted by the council on 26 November 1997.

### Implants

**Figure 1:**
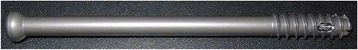
**Cannulated inside-out screw with flow geometry in thread.**

The test articles were modified Asnis screws (Stryker GmbH, Schönkirchen, Germany), 6. 5 mm in diameter, with a length of 60 mm. Modification of these titanium screws included additional holes on the side of the thread of each screw to allow the melted polymer to spread out (Figure 1). Test article A represented a thread of 10-mm length and test article B a thread of 20-mm length.

**Figure 2:**
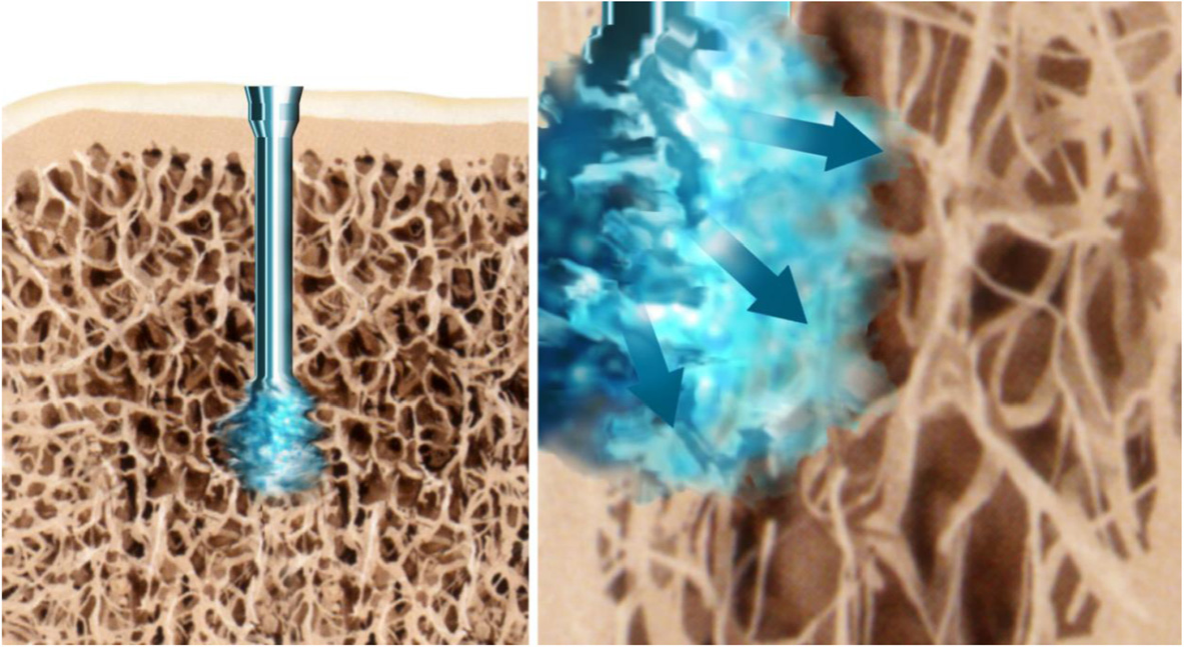
**Schematic view on the mode of operation of the melting in a SonicPin™ osteosynthesis.**

For augmentation, an inside-out (poly-L-lactide) PLDLA was used. The PLDLA pin consists of a polymer of poly(L-lactide-co-D,L-lactide) at a ratio of 70:30. Using ultrasonic energy, the fusion process binds a resorbable thermoplastic polymer device to the bone. The applied energy had a frequency of *20 kHz*, *an amplitude of 20 μm*, *and a power of 10 W* and was applied by a specific ultrasound applicator to the top of the pin. The ultrasonic signal of high frequency and low amplitude is applied resulting in a local melting of the pin tip. *Tissue damage because of the heat impact could not be found* [23,30]. The liquefied polymer flows out of the perforation in the screw and fills the lacunae of the cancellous bone (Figure 2). Immediately after stopping the use of ultrasound, the material solidifies and hardens, providing a three-dimensional anchoring.

### Surgery

The animals were operated under a combined anesthesia which included barbiturates and atropine. Each animal received analgesia preoperatively and also a prophylactic infusion of penicillin. After disinfection, an incision after disinfection on the medial side of the femur, proximal and partly over the knee joint, was made. The muscles were separated, and the implantation site was cleared. The two implants on each operation site were spaced 12 mm apart. A K-wire (3. 2 mm in diameter) was placed perpendicular to the bone axis. Drilling was conducted using a cannulated drill of 4.9-mm diameter, followed by rinsing with saline to remove debris. For the actual augmentation process, polymer was placed into the screw before implantation, and the screw was manually inserted into the drilling hole. After putting the ultrasound device on the polymer, activation of the ultrasound device led to a melting of the polymer, which was inserted under constant pressure. The contralateral screws were not augmented (control group). Afterwards, the incision was closed by suture, and the operated legs were not restrained. After surgery, a mediolateral radiograph was taken.

Three time periods for observation were chosen (*n* = 4 sites per group and time period): 4 weeks (4 animals), 8 weeks (4 animals), and 12 weeks (4 animals). In a paired approach, four test articles were implanted in each animal (Table 1): A (thread of 10-mm length) and B (thread of 20-mm length), both augmented with polymer; the contralateral hind limb was used for both screws A and B, applied without augmentation (control group).

**Table 1:** **Study design**

		**Time period**		
**Test article**	**Process**	**4 weeks**	**8 weeks**	**12 weeks**
Test article A, test	Augmented	4	4	4
Test article A, control	Non-augmented	4	4	4
Test article B, test	Augmented	4	4	4
Test article B, control	Non-augmented	4	4	4
Total number of sites		16 screws	16 screws	16 screws

Upon termination, the implant sites were macroscopically inspected and graded for inflammation. The histological comparison of the implantation sites was used to examine the biocompatibility of the SonicFusion™ inside-out technology.

### Scoring

At the appropriate termination interval, after sedation, the designated animals were euthanized with a lethal injection of barbiturates.

The implant sites were inspected and graded for inflammation macroscopically. The color and consistency of the tissue at the implanted sites was observed and recorded using a semi-quantitative scoring system (Table 2).

**Table 2:** **Semi-quantitative scoring system for macroscopic inflammation reaction**

**Reaction**	**Grade**
None	0
Light	1
Moderate	2
Marked	3
Severe	4

For histological analysis, the femora were harvested and samples of the implant sites and surrounding bony tissue were collected and fixed in 10% neutral formalin. The samples were dehydrated in alcoholic solutions with increasing concentrations, cleared in xylene, and embedded in resin. The embedded samples were cut along the longitudinal axis of the implant and stained with modified paragon. A semi-quantitative evaluation of the local tolerance was performed in compliance with ISO 10993-6, which included the evaluation of fibrin, necrosis, tissue degeneration, signs of infection, inflammatory reaction (polymorphonuclear cells, eosinophilic polymorphonuclear cells, lymphocytes, plasma cells, macrophages, giant cells), fibrocytes, fibroconnective tissue, and neovascularization. Particular attention was paid to the thermal effects of the augmentation procedure on the surrounding tissue. The performance of the augmented/non-augmented processes was assessed qualitatively and semi-quantitatively assessed by analyzing osseointegration and bone neoformation, osteoblasts and osteoclasts, fibrous tissue, bone remodeling, signs of infection, and material degradation.

**Figure 3:**
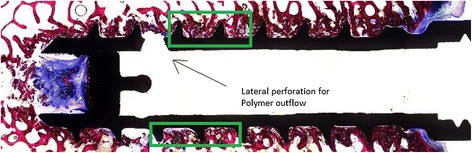
**Histological view of augmented screw (test article B).**

**Figure 4:**
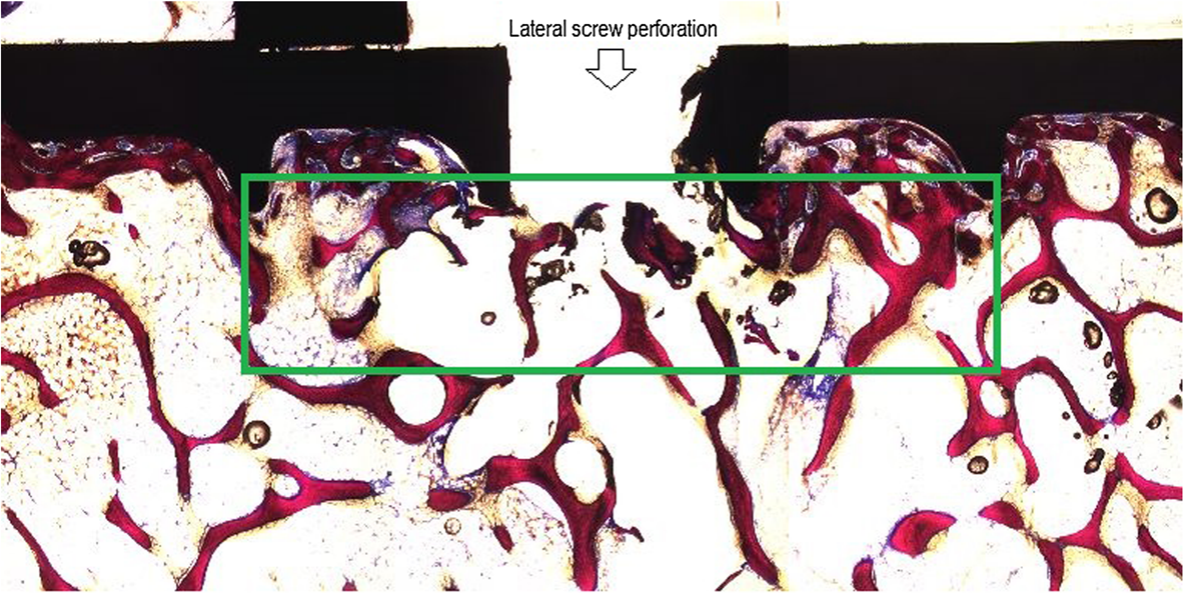
**Histological view of augmented screw (test article B) next to lateral perforation for polymer.**

The BIC was examined in a blinded manner by digitalizing and examining the slides with Zeiss Axio Scope microscope (Carl Zeiss microscopy GmbH, Jena, Germany) equipped with a color image-analysis system. The surface of the implant (screw or polymer) and the surrounding tissue was measured quantitatively in percent (Figures 3 and 4).

### Statistical analysis

Statistical analysis of the histomorphometrical data were performed using a Mann-Whitney test (SPSS version 15.0, USA).

## Results

### Macroscopic scoring

All screws (augmented and non-augmented) could be implanted without technical complications.

The macroscopic approach showed slight-to-moderate signs of edemas in the 4-week group. The 8- and 12-week group did not exhibit any noticeable problems (mean points in semi-quantitative macroscopical scoring 1.75 vs. 0.0 vs. 0.25, (acc. Table 2)). There were no signs for infection or inflammation in any group. Significant differences were not found by macroscopic scoring.

### Histological scoring

In the 4-week group, the non-augmented screws (*A and B*) showed a good level of osseointegration, despite occasional interpositional fibrous tissue. The non-augmented screw *B* was slightly better osteointegratedthan screw *A*. Observation of the augmented screws showed a similar polymer spread through the neighboring bone lacunae in both screws. In both augmented screws, the polymer or associated thermal effects resulted, in evidence of non-living and living bone embedded within the polymer, with slight signs of focal bone marrow fibrosis and absence of necrosis and bone absorption. Around the polymer, the peripheral bone showed signs of healthy and active bone tissue. The augmented screw *B* was slightly better osseointegrated than augmented screw *A*. No evidence of inflammation or infection could be observed.

In the 8-week group, the non-augmented screws (*A and B*) showed a good level of osseointegration, despite occasional interpositional fibrous tissue. After 8 weeks, both of the non-augmented screws showed a similar osseointegration. Slight bone ingrowth into the lateral screw perforations could be observed. In both augmented screws, the polymer spread into the surrounding lacunae of the bone. In comparison to the 4-week group, fewer inflammatory cell and less osteoblast activity could be found. In screw group A, the bony trabeculae embedded within the polymer showed discreet signs of atrophy. Around the polymer, the peripheral bone showed signs of healthy and active bone tissue. No significant evidence of inflammation or infection could be observed. Polymer degradation was not found after 8 weeks. Augmented screw *B* was slightly better osseointegrated than augmented screw *A*.

In the 12-week group, the non-augmented screw *A* showed slight-to-moderate signs of peripheral inflammatory reactions (fibrin, macrophages, osteoclasts), while the non-augmented screw *B* exhibited a better osseointegration without significant pathological changes. In both augmented screws, the polymer spread into the surrounding lacunes of the bone. After 12 weeks, the augmented screws showed less inflammatory reaction than after 4 or 8 weeks. Good osseointegration with some interfacial fibrous reactions could be observed.

The thermal effect on the bone marrow was no longer visible. Living and non-living osteocytes embedded in the polymer could be observed as well. Around the polymer, the peripheral bone showed signs of healthy and active bone tissue. No evidence of inflammation or infection could be observed. After 12 weeks, there were no signs of a polymer degradation. The augmented screw *B* was slightly better *osteointegrated* than augmented screw *A*.

### Bone-to-implant contact (BIC)

At no point in time were there any statistical differences in the non-augmented group. In the augmented group, there were no significant differences in the BIC of the screw itself and the surrounding tissue. The analysis of the BIC with the polymer showed significant differences in the 4- and 8-week group concerning the comparison of screw A and B (*p* = 0.021 and 0.019, respectively). The comparison in the 12-week group of screws A and B with regard to the BIC with the polymer was not significantly different (Table 3).

**Table 3:** **Bone-to-implant contact (BIC) in percent**

**Time period**	**BIC with screw material (non-augmented) (%)**	**BIC with screw material (augmented) (%)**	**BIC with polymer material (augmented) (%)**
4 weeks	B: 18.5	B: 17.8	B: 21.8
	A: 11.9	A: 9.6	A: 55.2 (significant)
8 weeks	B: 20.5	B: 17.7	B: 42.2
	A: 23.9	A: 17.3	A: 74.5 (significant)
12 weeks	B: 30.2	B: 42.3	B: 65.8
	A: 19.8	A: 39.0	A: 58.9

## Discussion

All screws as well as the augmentation could be applied safely. The macroscopical analysis showed a slight edema in the 4-week group, due to surgical trauma. There were no signs of infection or inflammation in any groups.

At all healing intervals, whether with or without augmentation, histopathological analysis showed that screw B (thread of 20-mm length) was slightly better osseointegrated than screw A (thread of 10-mm length), due to higher contact area because of the longer tread.

The non-augmented screws were considered as locally tolerated as well. The desired effect of polymer flowing out of the screw was confirmed in all cases of augmented screws. The extruded polymer was always in accordance with the polymer located in the screws. If bony tissue was completely embedded in and surrounded by polymer, rare non-living osteocytes and a slight bone marrow fibrosis could be observed. After a healing time of 12 weeks, however, complete healing and renewal of the adjacent medullar tissue was observed. No significant signs of necrosis, bone resorption, or biodegradation could be seen around the polymer.

All in all, the osteointegration of screw B (thread of 20 mm) exhibited overall the best results. The overall anchoring potential of the augmented screws outperformed that of the non-augmented screws, and the limited local thermal effect of the melting process was not significantly detrimental to the adjacent and limited portion of bone tissue involved.

The analysis of the BIC was uncertain due to the difficulties of being able to clearly identify the limits between the living and non-living bone in contact with the polymer. The BIC of the screw itself with the bony tissue showed no significant differences. But as seen in Table 2, the BIC of the polymer with the surrounding bone is significantly higher than the BIC with the bare screw to the bone (*p* = 0.038). Furthermore, the comparison of the augmented screws A and B to each other showed significant differences in the 4- and 8-week groups.

In their study, dealing with cement augmentation in pedicle screws, Sarzier et al. could show that the more osteoporotic the bone, the more significantly different is the anchoring of the screws [23,31]. A good bone-to-implant contact led to higher pullout forces. Paech et al. could prove in a biomechanical laboratory test that polymer-augmented lag screws in the treatment of femoral neck fractures exhibited a better protection against a cut out failure in osteoporotic bones [32]. The augmented screws exhibit a particular advantage in the cases of osteoporotic bones and can therefore also improve the stability of a screw osteosynthesis in these cases.

Kock et al. reported about an *in vitro* study of a polymer-augmented screw fixation with good pullout and torque forces, when a conventional screw is applied in a drilling hole, that was filled with a new resorbable polymer (on the basis of alkylene bis(oligolactoyl)methacrylates) beforehand [33]. Whether this testing scenario can be implemented in clinical practice is yet to be shown.

Concerning the BIC, Huang et al. could demonstrate in a laboratory study that a higher BIC is significantly correlated to a higher primary stability [34]. For the animal trial, a higher primary stability of augmented screws can be postulated if a higher BIC can be observed as was the case in our study. *Mechanical tests have yet to follow before clinical test can be performed*.

## Conclusion

Screw augmentation by resorbable, ultrasound-activated polymers is safe and biocompatible. No thermal effects on the adjacent bone were observed, while the bone-to-implant contact can be increased by this technique. In cases of osteoporotic fractures, this technique might provide a higher anchoring potential with a resorbable material.
